# Shifting vulnerabilities: gender and reproductive care on the migrant trail to Europe

**DOI:** 10.1186/s40878-018-0089-z

**Published:** 2018-07-06

**Authors:** Vanessa Grotti, Cynthia Malakasis, Chiara Quagliariello, Nina Sahraoui

**Affiliations:** 1 European University Institute, Robert Schuman Centre for Advanced Studies, Via Boccaccio 121, 50133 Firenze (FI), Italy; 2 École des Hautes Études en Sciences Sociales, Centre d’études des mouvements sociaux, 54-56 Boulevard Raspail, 75006 Paris, France

**Keywords:** Migration, EU borderlands, Sexual and reproductive health, Pregnant crossings, Gender

## Abstract

The reproductive care of pregnant migrants entering the European Union via its Mediterranean borders represents an under-examined topic, despite a growing scholarly emphasis on female migrants and the gendered aspects of migration in the past three decades. This article uses ethnographic data gathered in Greece, Italy, and Spain to examine pregnant migrants’ experiences of crossing, first reception, and reproductive care. We discuss our findings through the conceptual lens of vulnerability, which we understand as a shifting and relational condition attributed to, or dynamically endorsed by, migrant patients within given social contexts and encounters. We focus on two principal aspects of migrant women’s experiences. First, we shed light on their profiles, their journeys to Europe via the three main Mediterranean routes, and the conditions of first reception. Through ethnographic vignettes we examine the diverse ways in which pregnant migrants become vulnerable within these contexts. Second, we turn to the reproductive healthcare they receive in EU borderlands. We explore how declinations of ideas of vulnerability shape the medical encounter between healthcare professionals and migrant women and how vulnerability is dynamically used or contested by migrant patients to engage in meaningful social relations in unpredictable and unstable borderlands.

## Introduction

Scholars have engaged with the feminisation of migration for three decades. While the socio-demographic profile of female migrants has changed, the share of women in international migration has remained stable since the 1960s (Dumitrou & Marfouk, 2015). The more recent emphasis on feminisation derives from the gendered lenses of analysis increasingly adopted since the 1990s (e.g., Donato, Gabaccia, Holdaway, Manalansan, & Pessar, 2006). The notion of the feminisation of migration highlights differences between women migrating for family reunification (Salih, 2003), women migrating for work in the domestic and care (Andall, 2000; Kofman & Raghuram, 2015; Salazar Parrenas, 2001; Schrover, Van Der Leun, Lucassen, & Quispel, 2008) or more highly skilled sectors (Dumitrou & Marfouk, 2015), or undocumented migrants or asylum seekers in transit (Alsaba & Kapilashrami, 2016; Freedman, 2012, 2015a, 2015b, 2016; Lund Rasmussen, 2015; Stock, 2011). Feminisation may have been overstated, but a gendered analysis unlocks aspects of contemporary migration that remain under-researched, such as reproductive healthcare on the move. Reproductive rights extend over the legal rules of access to care to the socio-culturally mediated and gendered interactions between women and healthcare personnel.

We focus here on reproductive healthcare in southern European borderlands; the notion of borderlands emphasises the specificities of ‘borderlands milieu’ rather than the border itself (Martinez, 1994). Women and children made up respectively 17 and 26% of Mediterranean arrivals in Europe in 2016^1^; i.e., just slightly less than half of the arrivals across the three main Mediterranean routes through Italy, Greece, and Spain. The proportion of women is at its highest in Greece; in 2016, they composed 21% of sea arrivals, compared to 13% in Italy and 8% in Spain. Whilst the post-2015 cohort of female Syrian refugees has benefited from a wider coverage, little in-depth research has been carried out on the experience of women’s particular crossing conditions across the array of migration routes. Issues such as contraception and sexually transmitted diseases, or pregnancy and childbirth are strikingly underexplored. Yet pregnant crossings often encapsulate complex social dynamics constitutive of international migration networks today.

Beyond academic circles, international non-governmental organizations have published reports on gender-based violence during migration (Alarm Phone, 2018; Amnesty International, 2016; Nobel Women’s Initiative, 2016). Such publications are based on testimonies and qualitative interviews carried out in host countries thus offering an outlet for marginalised voices (Parker, 2015; Yasmine & Moughalian, 2016). Associating gender with vulnerability within these works and within the humanitarian sector in general has been questioned; such a systematic correlation can obscure how male migrants face gender-specific vulnerabilities (Turner, 2016).

In this article, we examine the experiences of pregnant migrant women during their journey into Europe and their stay in EU borderlands through a conceptual lens that offers a critical and reflexive approach to the concept of vulnerability. We define vulnerability as an intersubjective relation which is fundamentally ambiguous and subject to transformation according to actors’ perspectives, shifting through scales of equivocal compatibilities (Butler, Gambetti, & Sabsay, 2016; Kelly, 2011). Migrant maternity care in borderlands becomes a ‘productive site’ of processual relations, which redefine those ascribing vulnerability to others (patients, relatives of patients, etc.) and those who receive, perform, or demand the label of vulnerability. Migrants’ vulnerabilities are produced within the circumstances of their journey and reproduced within their interactions with medical institutions – yet, rather than fixed categories, they constitute dynamics forms of social engagement, ‘shifting’ vulnerabilities. Therefore, vulnerability can be considered to be generative of and complementary rather than opposed to agency (Butler et al., 2016; Chavel, 2013). Vulnerability is socially produced, through people’s locations in hierarchical social orders and power relations and effects (Quesada, Hart, & Bourgois, 2011).

We will discuss the circumstances of the journey along the three main Mediterranean routes and the conditions of reception, particularly within encounters of care. Female research participants became vulnerable because of their exposure to social contexts, structures, and relations. We critically document these experiences but also perceptions and constructions of vulnerability, and the models of assistance offered to migrant women. We further emphasise the agency migrants demonstrated in response to their “induced” (Butler et al., 2016, p. 2) or ascribed vulnerability. Their agency indicates responses that suggest alternative modes of resistance and empowerment.

## Methodology

Based on long-term ethnographic research with rescue and care services catering to pregnant women arriving in Greece, Italy, and Spain, this paper sheds light on their distinct profiles, routes, and histories. We interviewed migrant women positioned along the documented/undocumented continuum (undocumented, asylum seekers, migrants with temporary permits, or recognized refugees). Over several months (July 2016 to August 2017), we examined the provision of maternity care, focusing ‘symmetrically’ on the perceptions, discourses, and practices of migrant women and their various caregivers in the context of their interactions (Kelly, 2011; Latour, 1991, 2007).

Our specific locations were the following: Athens, Greece; Sicily and Lampedusa, Italy; and Melilla, Spain.

Research in Greece focused on Athens. Malakasis conducted a multi-sited ethnography of maternity care in numerous health and residential facilities. Her main research sites were a) an independent Mother-Baby Centre in downtown Athens that subscribes to the midwifery model of care and has been providing maternity care and breastfeeding consultations to hundreds of migrants and refugees from Syria, Afghanistan, and a number of Middle Eastern and African countries since it opened in September 2016; b) a satellite clinic of a major transnational health NGO, funded by the UNHCR and established specifically to offer medical, including maternity, services to refugees accepted to relocate and apply for asylum in another EU member state and c) the outpatient department and the labour ward of a major public maternity clinic. In these settings, she observed the medical consultations and also the labour of pregnant migrants, always with the patients’ explicit consent. In addition, she conducted of ethnographic interviews with five Syrian women from November 2016 to July 2017 using the services of a Syrian-Greek female interpreter. Malakasis interacted with the women in the camps, squats, and NGO-run hotels and apartments where they resided. She encountered her Syrian participants in her capacity as ethnographer-volunteer at the Mother-Baby Centre, where she asked them to participate in the research.

In Sicily and Lampedusa, Grotti and Quagliariello worked primarily in maternity health services, where they observed reproductive health consultations (pregnancy, miscarriage, abortion request, contraception, infertility problems, etc.) of local as well as migrant women. Additional sites included Lampedusa’s public spaces, and particularly the harbor where migrants disembarked almost on a nightly basis. Quagliariello further processed medical records from 2013 to 2017, on the nationality, age, family situation and medical situation and needs of both migrant and local women. The research focused on English and French speakers from Nigeria, Ivory Coast, Cameroon, Mali, and Guinea Conakry. Quagliariello conducted 15 interviews with migrant women. Another ten interviews were conducted with health professionals (three gynaecologists, three general doctors and four nurses) at the Lampedusa health facility.

In Melilla, Sahraoui worked in two sites: a) the Centre for the Temporary Stay of Immigrants (CETI) and b) the public hospital. Data presented in this paper are based on fieldwork inside the Centre, interviews and participant observation of daily life. Interviews were conducted in Spanish and in Arabic by the researcher, with the support of an interpreter when needed for dialects. After obtaining administrative authorization, Sahraoui approached the Centre’s nurses. Ten interviews were conducted with healthcare professionals employed by an international NGO providing primary care inside the Centre. The researcher’s daily presence allowed for informal moments with healthcare professionals. Interviews were further conducted with 17 migrant residents, pregnant or recent mothers.

All data are anonymized, and we use pseudonyms for the selected quotes. The research methodology and particularly the ethnographic work with migrant participants has been subject to extensive review by the European Research Council Executive Agency (ERCEA) Ethical Assessment Committee.

## Migration and reproductive health in southern European borderlands: contextual insights

Maternity care is legally classified as ‘urgent care’ under humanitarian clauses in Greece, Italy, and Spain, meaning that a pregnant woman may access healthcare regardless of her legal status. In Greece, Law 4386/2016 stipulates free maternity care for all women of no means or insurance whatever their political or legal status. In Spain, the Real Decreto-Ley 16/2012 limited access to healthcare to individuals with legal residency, but pregnant migrants were a category exempt from this restrictive turn. In Italy, Law 189/2002, following previous legislation, grants access to maternity care to pregnant foreigners; if they reside illegally, they may request a temporary residence visa that covers pregnancy and the first 6 months of the child’s life.

Maternity wards, then, may be seen as sanctuaries, where immigration agents are barred and care professionals are under no legal obligation to report patients. Yet, despite these protective provisions, the translation of health policy into the underfunded, over-burdened reality of densely crossed borderlands presents a challenge to providers as well as beneficiaries.

Athens is a state capital, but we understand it as a borderland in its capacity as a waiting area of sorts to the wider physical and legal-political territory of the European Union, to which the overwhelming majority of migrants in Greece has wished to travel. Our own ethnographic observation and surveys verify this claim ("Poly mikro pososto prosfygon epithumei," 2017; Kάπα [Kapa] Research, 2016). Migrants in Athens focus on the administrative process of moving to northern or western Europe, and seek accommodation and care that fit their – ideally – temporary presence. The accommodation and care provided to them, at least during the period of our research, was of a highly provisional character, betraying the fact that governmental, NGO and other actors were also operating under the impression that migrants’ stay was be temporary. Some 60,000 refugees, of all nationalities, among the 1 million or so who have tried to use the country as transit since 2015, are currently in Greece.

Lampedusa is the southernmost Italian territory in the Mediterranean Sea. This island of approximately 6000 inhabitants is located 220 km away from Sicily and 113 km from Tunisia, the closest landfall to the island. Lampedusa is therefore the first Italian territory that migrants encounter along their journey from African continent. In the Italian reception system there are five First Aid and Reception Centres (CPSA or hotspot) solely geared towards registration and initial reception. One of these centres is located in Lampedusa. Although migrants should only stay a few days in the island, delays of several weeks are not uncommon before their transfer to Sicily. Its position makes Lampedusa a highly prominent and symbolic migration stopover, although the number of migrants who pass through are a fraction of those taken directly to Sicily and the mainland. In 2016, 11,000 of the 180,000 people who crossed into Italy were taken to Lampedusa, while 65% were brought to Sicilian ports.^2^ Most boats rescued and intercepted by the Italian navy are taken to the Italian port of Augusta, on the Eastern Sicilian coast.

In Spain, more than 8000 persons arrived by sea in 2016,^3^ but our discussion focuses on Melilla, a Spanish enclave in northern Africa, and, along with its sister-city, Ceuta, the only terrestrial border between the EU and the African continent. Surrounded by militarised fences on one side and the Mediterranean Sea on the other, Melilla has become simultaneously a passage and a barrier to international migration routes to Europe. Melilla is a middle-sized city of approximately 84,000 inhabitants and only 12 km^2^. Migrants arriving in Melilla enter EU but not Schengen territory. In this context, Melilla has become another of Europe’s antechambers, where migrants wait for their authorisation to be transferred to what locals refer to as the ‘peninsula’. As in Athens, the assumption of temporariness shapes the material conditions of migrants’ accommodation, but here too length of stay varies and can extend to several years.

## A gendered study of routes into southern Europe

The journey through these three trails, and particularly the absence of legal and safe passage, constitutes the first set of conditions that render migrant participants vulnerable to numerous perils. These include physical and sexual violence, but also subtler yet severe forms of distress, such as separation from loved ones or the inability to plan and control the circumstances of their journey. Entry into these three borderlands combines dangerous land and sea travel; however the Central Mediterranean route, which connects West and East Africa to Niger and Libya before entering Italy, is by far the deadliest in absolute terms (Black, Dearden, & Montes, 2017),^4^ and one along which our data highlight the highest levels of violence against women.

### The Central Mediterranean route

In Italy as a whole, the proportion of migrant and asylum-seeking women is consistently low, at about 10 %. In southern Italy, this low proportion drops slightly, with estimates varying between eight-9 % (International Organization for Migration [IOM], 2017). In Lampedusa, this proportion was even lower at 7 % of the total crossings; out of this small contingent, 5 % were pregnant. The largest proportion of women is from Nigeria, Eritrea, Ivory Coast, and Somalia. These figures reflect the two main routes used by migrant women from Sub-Saharan Africa to Europe through the Central Mediterranean route. The first route originates in West Africa and requires two or three international borders to be crossed before arriving in Libya (Nigeria-Niger-Libya, in the case of Nigerian women; Ivory Coast-Burkina Faso-Niger-Libya for Ivory Coast nationals). The second route starts in East Africa, and again requires women to cross two or three international borders before entering Libya (Eritrea-Sudan-Libya, in the case of Eritrean women; Somalia-Ethiopia-Sudan-Libya for Somali women). On the Western-Central route, the main place for the passage into Libya is Niger; on the Eastern-Central route, Sudan must be crossed to enter Libya.

Female patients in Sicily and Lampedusa normally travel longer than anticipated, from 2 months up to a year before reaching Libya. Those who can pay for all the stages of the journey upfront manage to move more rapidly to Libya and then to Europe. Those without enough resources must earn their passage, usually by working in transit countries before reaching Libya. This phenomenon is widespread among male migrants, often involved in harsh physical labour such as construction work. Women have fewer available options, in domestic work or the service industry, although employment conditions tend to merge labour with sexual requests and favours in trafficking spirals, which lead female employees to fall into prostitution rings from an early stage of their journey. The absence of kin or social networks and the strong dependence of women on men to broker milestones or ensure protection on the migration trail expose them to psychological, physical, but also sexual violence. This situation is particularly common during the last leg of the journey into Libya, where women are systematically subjected to sexual assault, both at the hands of the Libyan police and the smugglers (Amnesty International, 2016). The latter are described as the gatekeepers who can decide when (or whether) to allow their ‘clients’ to cross the Mediterranean into Europe.

Apart traveling alone or with female relatives, women also travel with male partners. Women often become pregnant as they are on the move, but frequently have to deal with the pregnancy alone. Pregnant women are often separated from their partners during the journey, as the latter get arrested or are forced to work for traffickers. In other cases, men left the country of origin to work in Libya prior to 2011; women joined them in Libya after a few years through family reunification policies. Yet the economic and political crisis in Libya forced these couples (who initially had planned to return home after gathering sufficient income) to flee to Europe.

Rose’s history encapsulates the plight of female African migrants as they travel to Europe. Rose originates from Kano, in northern Nigeria. She is 27 years old and has a 4-year-old son, whom she left with her aunt in Nigeria. This same aunt had taken care of her since she was very young. Her parents could not afford to raise their five children. Rose left school when she was 11, to start selling water at traffic lights and doing other small jobs. Her son’s father left her when she got pregnant. Rose decided to migrate to provide a better future for her son. Yet her money could only get her from Nigeria to Niger. There she encountered a Nigerian man who subsidised her journey farther north. It took them a month to enter Libya, where they remained for 6 months. As they tried a first departure to Italy, the Libyan police seized them. The man was arrested; she was forced to move to a migrant camp, along with other African women, until they could leave for Italy. By the time we spoke to her, Rose had lost contact with her travel companion for a month, and she did not know how to help him get out from jail. Rose was more than 5-months pregnant when we first met her. She thought that her pregnancy was the result of the relationship with her partner, with whom she had travelled with from Niger to Libya. However, she was not entirely sure, as she had been assaulted by two men in the migration camp in Libya. During our meeting at the maternity service of Lampedusa, Rose showed us several signs of cuts and burns on her right leg, then proceeded: ‘That’s what they did to me in Libya, as I did not have the money to get Kevin (her travel companion) out of jail. The signs of all that I have suffered are not only on my body but also inside me'.

### The Eastern Mediterranean route

While the journey to southern Italy renders migrants vulnerable to extreme forms of physical and sexual violence, Syrian migrants in Greece navigated circumstances that produced different forms of vulnerability – perhaps subtler yet psychologically debilitating and often with severe physical perils. At 37% of the 9461 sea arrivals in 2017, as of June 30,^5^ 47% of the 173,450 total in 2016,^6^ and 56% of the 856,723 total in 2015,^7^ Syrians form the largest national group that has been arriving in Greece. Further, our ethnographic observation confirms the UNHCR claim that Syrian women in their majority seem to travel with close relatives, as a nuclear family unit caring for children,^8^ and sometimes with elderly dependents on what is a highly perilous journey through a country at war, strewn with countless roadblocks controlled primarily by ISIS, but often by other forces. Each roadblock presents a new chance of being killed; each roadblock also requires a new bribe to be allowed to cross. After crossing the Turkish border, families are usually apprehended and spend at least one night in jail and several days in hiding, until they find a new smuggler to help them cross the Aegean. Entry into Greek waters and the subsequent arrival at a Greek island often brings significant relief and a sense of physical safety, but also signals the beginning of a drawn-out administrative process in order to be able to continue the journey into the mainland. Syrian refugees who participated in our research in Athens had arrived in the country before the EU-Turkey Statement^9^ went into effect on March 20, 2016, and the majority wished to continue their land journey through the Balkans towards Germany and other northern-western EU Member States. Through word of mouth – or, less often, official communication – these families heard about their two options: to apply for relocation to another EU country, or to apply for asylum in Greece. Most opted for the former, and resorted to the latter if they were not selected for relocation or allowed to apply in the first place.

During the indefinite period of waiting for the administrative process that would authorize their journey northwest, Syrian refugees were subject to physical hardship, but also to a sense of no control over their circumstances and of a lack of care on the part of people who held such control. The case of one of our interlocutors, Hala, demonstrates the harshness of living conditions. During the first days of 2017, Greek and international media were dominated by images of tents in refugee camps in Greek islands buried in snow. Yet even in camps where refugees lived in metal containers rather than tents, conditions were by no means comfortable. Throughout the time we interacted with her, from December 2016 to May 2017, Hala lived in a camp in the city of Athens. In January 2017, Hala was 8-months pregnant. She was sleeping on a flimsy mattress on the floor of her one-room living unit; next to her slept her husband and his teenage cousin. When all three of them were lying down, there was no space for their small heater, which would have posed a fire hazard if it had been lit while they were asleep anyway. Even as the outside temperature plummeted below zero, the electricity failed every few minutes, because the general power switch was tripping in response to the volume of use. Without the heater, the temperature in the container reached outside levels within minutes; the container’s walls felt frozen to the touch. If the power fails during the night, Hala told us, nobody switches it back on. She argued that camp workers were indifferent to the refugees’ well-being. She also blamed such indifference for the fact that both EU and Greek asylum officials took months to register her and process her papers, and deemed her ineligible for the EU Relocation Scheme without ever justifying this decision. She also attributed indifference to camp officials in Lesvos, the island of first arrival, who she said let food provisions sit and rot for weeks in storage before offering them to refugees.

The feeling of disregard on the part of people with the power to offer or withhold decent living standards and to further or impede their journey northwest was expressed by all Syrian interlocutors. Their daily lives but also the continuation of their journey and their long-term life chances hinged upon bureaucratic processes that were obscure, overdetermined, and random – refugees were seldom informed of the rules and mechanisms that yielded decisions central to their fate. Hala and her husband were never informed that the asylum process in Greece entailed the permanent confiscation of their Syrian passports; they interpreted this as the malicious or neglectful violation of their rights by specific bureaucrats, and spent several anxiety-ridden days toward the end of Hala’s pregnancy, before Malakasis sought the advice of a legal specialist and relayed to them the rules of the asylum process. Another one of our Syrian interlocutors, Hajer, was convinced that the social worker in charge of her medical file at the NGO-run hotel where she resided was deliberately withholding information from her. During one of our visits to her at the hotel, she asked the social worker on duty for her medical file to show it to us; when the woman replied that it was under the care of the social worker appointed to Hajer, who was not there at that moment, Hajer’s anger flared. ‘It’s my file; I have the right to ask for it,’ she told us in a curt, angry tone, punctuated with sharp gesticulations. And a bit later, in a slightly more pleading tone, conveying more despair than anger: ‘I feel like a prisoner’.

The lack of control over their circumstances, coupled with the conviction that the people who wield such control are indifferent to migrants’ well-being, fosters an intense sense of helplessness. Combined with the objective reality of substandard living conditions, it rendered the women who participated in our research vulnerable in a physical but also a psychological sense.

### The Western Mediterranean route

Similar tensions between long, perilous journeys through transit countries and long-winded, bureaucratically opaque conditions in borderlands occur farther west, in Spain’s borderlands with Morocco and the African continent. Unlike its sister-city, Ceuta, which receives mostly single male migrants, Melilla hosts lone female travellers and families. In September 2016, children and women represented 37% of the CETI’s residents. Most pregnant migrants came from Syria, Algeria and Morocco. By the time they crossed the border into Spain, pregnant refugees from Syria had been on the move for 4 to 5 years, having resided in different African countries. Stopovers usually included Egypt, Algeria, Mauritania, Mali, and finally Morocco. While in transit, the maternity care they accessed was private care for which they had to pay; at times at reduced rates in Algeria thanks to the sympathy of some doctors for Syrian refugees. Once in Melilla and after having been granted a resident’s card (that allows entering and exiting the facility) in the CETI, medical care was organised through an international NGO in charge of primary care the Centre’s residents. Primary caregivers channelled migrants to public health structures and their medical specialists, midwives and gynaecologists. In September 2016, 726 migrants were hosted at the Centre, among them 124 women. Over the course of 2015, 59 pregnant women stayed in the CETI in Melilla; in September 2016 around 20 women were pregnant and many more were travelling with young children.

Among women staying in the CETI, Syrian women constituted the largest group. Fadwa’s story illustrates their experience. Fadwa is a 25-year-old Syrian woman, who had left Homs 4 years before. She had spent most of her time since leaving Syria in a UN-managed camp in Lebanon. Fadwa was more than 8 months pregnant when we first met her in the inner courtyard of the CETI. Over the 8 months of her pregnancy, she and her husband had travelled thousands of kilometres through seven countries. From Lebanon to Turkey, from Turkey to Mauritania, then to Mali, after that to Algeria, then to Morocco, and finally to Melilla, another stop on the long journey to Europe, somewhere between Africa and the European Union. Fadwa and her husband did not have enough money to pay for their passage to Europe through Turkey, so they had to attempt a longer route. Fadwa’s husband did not manage to enter Melilla at first, so Fadwa spent several days in the CETI on her own before he managed to join her, during which she was particularly worried since she did not know if he would manage to enter the Spanish enclave at all. Fadwa was not the only Syrian woman in this situation in the centre. Families could rarely cross the border together, and usually women made it first to the Spanish side. Women waited for their husbands, or at times brothers, for days or weeks, as the duration of these separations resulted from on an opaque mix of access to information and contacts, financial capacity for bribes, and circumstances at the border on the day of the attempted passage. The gendered workings of the border passage separated families, created vulnerabilities, and resulted in specific challenges both for the men who stayed stuck and the women who, like Fadwa, had to go through first reception without knowing if and when the rest of the family would manage to cross the border.

A few days after she had arrived in Melilla and before her husband had joined her, Fadwa had not yet received the pregnancy booklet that pregnant women are given upon their first visit to the midwife. Due to the characteristics of her journey and the obstacles still lying ahead, Fadwa did not expect to access specific maternity care upon arrival (she did later) and the many uncertainties that characterised her situation worried her more than the pregnancy that followed its course. She had seen gynaecologists over the past months in Mauritania and Morocco, and each time she had to pay out of pocket. Fadwa knew she was expecting a baby girl, her first child. Yet this was her second pregnancy. When she lived in the refugee camp in Lebanon, she gave birth after 6 months of pregnancy, and her premature baby did not survive. In the CETI, Fadwa found living conditions quite difficult, though a little better than what she had experienced during the journey. Each room had four bunk beds over a couple of square meters, and the occupancy rate exceeded eight persons when the women had small children, given that the latter slept in their mothers’ beds. Upon arrival, Fadwa was assigned the higher bed of the bunk beds, despite being 8 months pregnant. Each trip to the toilets, situated outside the room and a couple of meters away, turned into an acrobatic performance, which she mimed as she described it. Fadwa’s situation was thus not met with an institutional response to the specific vulnerabilities produced by her pregnancy in the context of a long and tiring journey; this example illustrates how inadequate material conditions exacerbated the gendered difficulties that her journey entailed.

Rose, Hala, Hajer, and Fadwa all faced specific gendered challenges on the migration trail to Europe. While difficult material conditions, stressful administrative uncertainties, and fragmented or hardly accessible healthcare constituted challenges shared by all asylum seekers, the lived experiences of women differed throughout the journey as well as in reception facilities along gendered lines of division. The following section provides insights into women’s reproductive care at the border by looking ethnographically into the gendered spaces, practices, and experiences of migrant maternity care.

## Productive vulnerabilities: constructions of and responses to vulnerability in spaces and relations of care

### Entering care spaces: being framed as vulnerable

Pregnant women receiving maternity care in EU borderlands flee violent conflict, political unrest, and poverty. They further face the loss of kinship or other support networks torn apart during the journey (separation by the police or traffickers at the border; administrative mistakes in reunification processes). Upon arrival, they are considered vulnerable because of their pregnancy, and are thus guaranteed access to healthcare facilities. We focus here on migrants’ reproductive care in borderlands and how vulnerability is produced discursively and performed within the practices of care.

Most pregnant women who arrive in southern Italy are young. Women we met in Lampedusa were between 17 and 35 years of age. Their level of education and social background varies greatly. In the Lampedusa health service, they are primarily offered pregnancy ultrasounds and consultations on the possibility of terminating pregnancies resulting from sexual violence during the journey. Contrary to mainland Sicily, pregnant women in Lampedusa are meant to be transferred farther north for health reasons before other patients. Yet their meeting with Lampedusan doctors represents a crucial milestone in their care trajectory in Italy, as this first check-up puts them in the national healthcare system by allocating them their personal health code, which allows free access to any hospital in Italy. This first examination also establishes the pregnancy stage; this information determines subsequent assistance and the legality of abortion requests in Italy. The health records, therefore, made by the doctors in Lampedusa and mainland Sicily guarantee medical care beyond first reception. Doctors we interviewed highlighted their power and responsibility to define the ‘health destiny’ of their migrant patients.

This awareness leads to a creative use of national medical protocols based on personal moral and ethical motivations. Doctors may elect to prescribe to pregnant migrants patients additional examinations (for instance, extra blood tests and screenings usually intended for high-risk pregnancies), whose information compensates for the lack of clinical data on the first months of pregnancy. Most migrant women rescued along the central Mediterranean have no documented medical history, and thus have their first ultrasound and blood test only once they reach Italy. On the one side, this hypermedicalisation links to an attitude of ‘defensive medicine’ wherein doctors perform additional tests to assess maternal and foetus risk in the absence of information obtained directly from patients (also due to language barriers). Yet this tendency also denotes the desire to provide the best possible care, the broadest possible protection and support. Doctors usually described pregnant migrants as extremely vulnerable psychologically, but also socially and materially. The resulting representation is not only one of people who need help, but also one of people who are harmless, unlike the representation of male migrants, especially Muslims. Another common discourse represents African women as victims of gendered exploitation, such as sexual trafficking. According to these imaginations, doctors’ feelings mostly related to the theme of pity and compassion (Fassin, 2007), which fostered the choice to take care of these women not only as ‘gynaecological patients’, but also as persons who had lived through an extremely difficult journey and are at the risk of ending up in the prostitution market in Europe. The request for psychological support – beyond the clinical needs related to pregnancy – exemplifies this sympathetic attitude.

In Athens, medical professionals in the public and NGO sectors perceived Syrian migrants as vulnerable firstly because of pre-existing cultural stereotypes which persisted because of the brief and structured interaction. Most midwives and doctors did not interact with migrant patients long enough to be able to detect the agency that women exercised within their culturally conditioned gender roles. The Syrians’ frequent childbearing, for example – most women we encountered in care spaces had a minimum of three children – was seen as imposed upon women not autonomous enough to make their own reproductive choices. Yet we argue that this orientalist perception of Syrians as women who have passively surrendered their reproductive agency to the authority of their husbands was reinforced by the almost complete lack of linguistic interpretation in Greek hospitals, where patients stood voiceless in front of over-worked medical personnel scrambling to get their history or symptoms. Unable to converse with women, midwives and doctors often reiterated the idea that pregnant migrants were passive to the point where they relied on their husbands to remember the first day of their last period (the first piece of information solicited by medical professionals in the beginning of a consultation). Yet our own observations, in the premises of health NGOs, where linguistic interpretation was provided, strongly belies this impression. Almost all women whose consultations we observed were in full knowledge of their reproductive history and symptoms.

In Athens, apart from public hospitals, maternity care is provided by a network of health NGOs (in their own premises or via mobile units that visit camps in the wider Attica region), but also in a small centre downtown, established and staffed by midwives and lactation consultants who adhere to the midwifery rather than the biomedical model of care. Indeed, a large part of antenatal care in Athens is provided by the humanitarian sector, which refers women to public hospitals for their diagnostic tests (NT scan, B-mode or Doppler ultrasound, etc.), as well as blood and urine tests. Women bring their results back, and NGO doctors monitor them. Toward the end of their pregnancy, they urge them to open a medical file at a public hospital, so that hospital health personnel are familiar with their history when they go into labour. Alternatively, pregnant patients can be monitored in public hospitals throughout their pregnancy. Whether antenatal care is provided by NGOs or by the public sector depends largely on where they are channelled by the officials in charge of their overall accommodation (social workers in camps or NGO-run hotels, or activists who have organized the city’s refugee-housing squats). Migrants’ care inscribes onto an existing and intensifying divide between a medicalized model of care and one that views pregnancy and labour as physiological processes requiring medical intervention only in the case of emergency complications. This divide and the way migrants navigate it, forming alliances in response to the vulnerabilities they face, is discussed farther in this section.

In Melilla, most migrants giving birth in the maternity ward of the main hospital are not international migrants, the CETI’s residents, but Moroccan women who have either crossed the border temporarily, or reside in Melilla without documents. CETI residents are entitled to the same care as Spanish citizens; in practice, they received basic provisions, such as medical visits, but not ‘optional’ forms of care (e.g., birthing classes).

Fadwa’s experience followed established care paths for female residents at the Centre, facilitating a documented journey through local healthcare structures. When her labour started, the CETI paid for her taxi transfer to the hospital. There, she gave birth naturally, stayed for less than 24 h as most CETI women, and received good, as she said, care. When we visited her in the Centre afterward, her newborn was tightly wrapped into a cotton piece of cloth, and slept on the bed. The Centre had no cradles; babies slept in bed with their mothers, who remained half-asleep throughout the night from fear of harming the baby during their sleep. In the room, several women sat on the floor around Fadwa; they were chatting, and a couple of children played by going up and down the bunk beds. One of the women prepared some tea, using a kettle that she carefully dried, and put back in its packaging, after she had poured tea in plastic cups. CETI residents were not allowed to boil water or even bring food in the rooms; hygiene and safety first, claimed the social workers. Fadwa’s newborn was not the only baby in the room; another woman held a 3-month-old baby born in Morocco before she had arrived in Melilla. Impatient to leave the Centre and to continue her journey, Fadwa did not wish to receive more medical care.

Maternity care for pregnant migrants in Melilla differed according to their diverse ways of lacking documentation. The migration journey and language barriers did impact the continuity of care and the hierarchical character of the medical encounter across the board. However, while CETI residence produced an administrative acknowledgement of pregnant women’s health-related vulnerability, the undocumented status of Moroccan women, who were legally only entitled to emergency care, fostered suspicion rather than recognition. The articulation of migration and healthcare regimes thus created different contexts for pregnant women on the move and facilitated or restrained the access to care beyond the practices of individual healthcare professionals.

### Experiencing vulnerability and displaying agency within care spaces

In this section, we shift the focus from the ascription of vulnerability to pregnant migrants to the ways in which they ‘become’ vulnerable and the responses they display within care encounters. We focus mostly on the effects of the language barrier and of the hegemonic, biomedical model of maternity care.

In Athens, state medical personnel’s best intentions are curtailed by a major deficiency, which is the first thing most mention in relation to migrant patients: the lack of interpretation services, which leaves them unable to elicit migrants’ medical histories, inform them of their options or rights, or communicate with them during labour. Similarly, women interviewed in Lampedusa and Melilla were often dissatisfied with interpretation during the medical encounter; many complained that the interpreter was either not always present or, when present, unwilling to translate their questions to the doctor. This language barrier limited communication and de facto exacerbated the power relationship that characterises any doctor-patient interaction.

Women in the CETI, who had entered the regular network of care, were offered several options, such as an epidural during labour. Yet other undocumented women in Melilla were not given the same options, because their pregnancy was not at all or partially monitored. Moreover, care itself constituted a source of concern that was hardly voiced with healthcare professionals, since all women interviewed aspired to travel to the peninsula at the earliest possible date, a need that took precedence over medical concerns. Maternity care became highly politicised, as transfer to the peninsula was prohibited after the eighth month of pregnancy; CETI officials argued that boat companies refused to board pregnant women who might go into labour during transfer and force the boat to return. The rule upset pregnant CETI residents, because a) they considered its implementation arbitrary and b) it prolonged their stay in Melilla for at least 3 months, as they had to travel after the birth. Language difficulties prevented them from voicing their concerns and getting clear answers, but they nevertheless strove to crosscheck any information they gathered. Consequently, women came to see medical care as a potential obstacle to their migratory trajectory, thus associating the medical encounter with suspicion and anxiety.

The lack of communication discussed above exacerbates the power relationships inhering in the medicalization of childbirth. The medical care of Hajer, our interlocutor mentioned in the previous section, encapsulates pregnant patients’ routes along the network of maternity care in Athens and illustrates the challenges faced within these highly medicalized care spaces. Yet her case also speaks to the agency pregnant migrants exercise within circumstances that render them vulnerable.

Hajer’s prenatal care was provided by two health NGOs, one at a camp where she resided briefly and one in downtown Athens; by a public maternity hospital where she was taken by the social workers of a third NGO, which housed her; and by a small centre in downtown Athens which offers midwifery services to migrants. Hajer had two older children, at the time 9 and 11; her first birth had been a C-section, but she had delivered her second child naturally. Given her one C-section, however, the public hospital where she was taken for prenatal appointments refused to consider natural birth; Hajer was asked to sign a paper that she refused the C-section on her own volition, and therefore was responsible for seeking another medical facility to deliver. Hajer sought the support of a midwife she had grown to trust, who advised her to not go to the hospital until she was well into labour and almost fully dilated. Hajer concurred, and the midwife joined her at the start of her labour pains, and called an ambulance for her when she was almost fully dilated. She directed the ambulance to the maternity clinic of a different public hospital; upon arrival, Hajer’s water broke, and she delivered naturally less than an hour later. Hajer’s case demonstrates the diverse and often clashing agendas that determine the maternity care migrant women receive and the control they are able to exercise. Migrant women enter contexts of pre-existing, complex relations and divides – between activists, NGOs and the public sector; and between health professionals who favour different medical norms. Within these circumstances, however, migrant patients exercise agency that belies the equation of vulnerability with passivity. Hajer’s story exemplifies the *productive* aspect of vulnerability – in this case, the alliances pregnant migrants form and the way they deploy them to take control of their reproductive outcomes.

Many migrants we interviewed in Lampedusa defined their pregnancy as unwanted. They associated it with violence suffered in Libya – not with unprotected sex in previous stages of the trip. This highlights how events that occur in Libya inflict trauma on the lives of women. During interviews with gynaecologists, migrants often defined pregnancy as suffering added to the physical and psychological abuse experienced during migration. Therefore, they expressed the need for immediate termination. Despite their determination, access to voluntary termination of pregnancy was not always possible. According to the Italian law (194/1978), the request for VTP must be communicated to the gynaecologists before the end of the third month of pregnancy. Therefore, a request for an abortion is only possible for migrants who arrive in Italy before this deadline. Furthermore, Lampedusa’s outpatients’ clinic did not have a delivery room for childbirth, nor a neonatal intensive care unit or operating room for surgical terminations. After initial health checks performed by local obstetric gynaecologists, pregnant patients were transferred by medical helicopter or by boat (and then by bus) to other hospitals in Sicily, either in Palermo or in Agrigento. The helicopter transfer takes an hour, and is only offered to migrant women in their final months of pregnancy. The rest are offered transfer by boat (and then by bus), which takes about 12 h. Thus, VTP for migrants who arrive in Lampedusa with a pregnancy of less than 3 months is *endangered* by bureaucracy, which is necessary in order to arrange transfers to Sicily. Women arriving directly in East-Southern Sicily (Augusta harbour) are transferred either to Catania or Siracusa, depending on hospitals’ availability. Furthermore, it is necessary to remember the general difficulty of access to abortion in many hospitals in Sicily where, among doctors, conscientious objectors represent 87.6%.

In the three contexts we explored, pregnant women voiced their concerns and took decisions against the background of different opportunity structures, even if a shared linguistic challenge tended to disempower them in the medical interaction. The women we met made their priorities clear, for instance refusing that pregnancy had to lead to immobility in Melilla, requesting a VTP in Lampedusa, or opposing a C-section in Athens; they nevertheless faced administrative and bureaucratic barriers that challenged their reproductive rights and agency.

## Conclusion

Intimacy, safety, silence, and certainty are fundamental needs, which women in transit toward Europe must often do without for years on end. This article outlined the routes taken by women on the move and the challenges they faced during the journey along the three main Mediterranean routes and upon arrival in European borderlands. We analysed their experiences of the journey and maternity care upon arrival through the lens of vulnerability.

We defined vulnerability as an intersubjective relation, fundamentally ambiguous and dependent on actors’ perspectives. We demonstrated that, in the context of maternity and reproductive care, migrant patients a) are constructed as vulnerable, b) become vulnerable because of the circumstances of their journey and their care encounters, and c) respond to (the label of) vulnerability with forms of agency that challenge gendered, paternalistic modes of institutional protection.

In “A gendered study of routes into southern Europe” section, we demonstrated that the circumstances of the journey along the three main Mediterranean routes as well as the women’s efforts to continue their journey after arrival to these borderlands presented a multitude of gendered vulnerabilities. These included severe sexual and physical violence in the Central Mediterranean Route and less brutal yet significant forms of distress in the Eastern and the Western Routes; the latter include the severing of social and kinship ties, the dismal conditions of life in hotspots and camps, and the powerlessness and uncertainty within the administrative and bureaucratic process necessary for the continuation of the journey.

Pregnant migrants arrived in borderlands where, despite the high value placed on maternity, resources are scarce and well-meaning caregivers are often fatigued and exasperated. Our article outlined the structural impediments to reproductive care and rights in these sites. Yet, we also demonstrated how vulnerability but also perceptions of vulnerability were constructed via a combination of these structural factors and pre-existing, cultural and gendered stereotypes. Within this context of structural, socio-culturally mediated and gendered constraints, migrant patients exercise agency through the alliances they form, the help they seek, and the decisions and demands they make.

## Abbreviations

CETI: Centre for the Temporary Stay of Immigrants

## Footnotes

1 Retrieved from https://data2.unhcr.org/en/documents/download/53447

2 Source: Agrigento Prefecture - Immigration Police Office - CPAS Lampedusa.

3 Source: Retrieved from https://www.iom.int/news/mediterranean-migrant-arrivals-top-363348-2016-deaths-sea-5079

4 This international crossing is indeed ranked the deadliest in the world (International Organization for Migration [IOM], 2017).

5 Retrieved from https://data2.unhcr.org/en/documents/download/58495

6 Retrieved from https://data2.unhcr.org/en/documents/download/53447

7 Retrieved from https://data2.unhcr.org/en/documents/download/46811

8 Retrieved from https://data2.unhcr.org/en/documents/download/58495

9 This legal agreement between the EU and Turkey stipulates that the latter attempts to halt refugee flows to Greece and receives back people who did manage to cross. As a result, people arriving in Greece after it went into effect are not permitted to cross into the mainland, but are rather held in the islands pending their return to Turkey.
